# When Rituals Fail: Rationalization, Bayesianism, and Predictive Processing

**DOI:** 10.1002/evan.70020

**Published:** 2025-11-05

**Authors:** Ze Hong

**Affiliations:** ^1^ Department of Sociology University of Macau Macau China

## Abstract

Why do rituals persist in human societies despite their frequent and observable failures to produce intended outcomes? This paper advances a two‐part argument to explain this resilience. First, at the individual level, I argue that belief in ritual efficacy is maintained through Bayesian‐rational processes, where the invocation of auxiliary hypotheses absorbs disconfirming evidence and shields central beliefs from significant revision. Importantly, such protection is not complete. Each failure produces a small but non‐zero erosion of individual confidence. Second, I address the resulting population‐level puzzle: why does such incremental doubt not accumulate into widespread skepticism and the eventual collapse of ritual systems? I argue that social features and informational dynamics (e.g., memory biases, the underreporting of failure, pluralistic ignorance) as well as the protective “design” of rituals themselves systematically inhibit the aggregation of doubt across individuals and generations. By linking individual cognition with population dynamics, this account explains the remarkable resilience of ritual systems in the face of persistent empirical failure.

## 1 Introduction

Throughout history and across societies, humans have always attempted to achieve desirable outcomes such as hunting success, bountiful harvests, good health, harmonious relationships, and protection from danger through various actions like sharpening weapons for hunting, applying irrigation techniques for farming, consuming nutritious diets for health, and building shelters for safety. These instrumental actions make sense from both cognitive and evolutionary perspectives. Cognitively, such actions are reinforced when the desired outcomes follow, consistent with principles of operant conditioning [1]. Evolutionarily, actions that bring tangible benefits often confer a fitness advantage and are therefore favored by natural selection [2]. However, as extensive ethnographic and historical records reveal, many actions aimed at achieving specific objectives do not “work.” Praying to a deity, for instance, does not increase the likelihood of rain, nor does wearing a special charm render one invulnerable to bullets. Early ethnographic records indicate that individuals in traditional societies widely employed instrumental technologies that are ineffective—what we might term “magic” by modern scientific standards. But why do these practices persist?

This question has sparked considerable debate about human rationality, from early twentieth‐century scholarship to contemporary discussions [3, 4, 5, 6, 7, 8]. Early scholars such as Frazer [9] and Tylor [10] emphasized the instrumental nature of magical practices, theorizing their persistence in broadly psychological terms. This “intellectualist” approach suggested that magic practices reflected certain types of faulty causal reasoning, where practitioners mistakenly attributed success to the efficacy of their actions. Later scholars, however, largely abandoned this intellectualist perspective, critiquing it for oversimplifying indigenous practices and ignoring their cultural and social contexts. Instead, they favored symbolic and functionalist interpretations, arguing that magic practices are not primarily about achieving concrete ends but rather serve (often hidden) social functions or carry only symbolic significance, such as reinforcing social cohesion, expressing cultural values, or maintaining hierarchies [11, 12]. These interpretations often sought to protect indigenous peoples from accusations of irrationality, reframing magic as symbolic or socially functional rather than “incorrect” science [13].

In a series of publications, my colleagues and I have argued for the foundational importance of the intellectualist perspective, asserting that the instrumentality of magic and divination remains critical across diverse ethnographic and historical contexts [4, 14, 15, 16, 17, 18, 19]. We contend that confidence in these practices is shaped by domain‐specific cognitive preferences and biases in information transmission, aligning with cognitive approaches advanced by scholars such as Horton [20], Jarvie [21] and Boyer [22], who underscore the practical and causal reasoning underpinning ritualistic behaviors.

Building on this tradition, in this paper I examine how individuals and communities explain ritual failures in ways that preserve confidence in their efficacy. Rather than dismissing unsuccessful rituals outright, participants often invoke additional explanations to reconcile discrepancies between expected and observed outcomes [5]. While such ad hoc rationalizations are pervasive, I argue that the cognitive consequences of ritual failures should not be overlooked as these failures can sometimes exert a nontrivial impact on beliefs. Though rarely explicit in ethnographic records, most ritual failures likely diminish confidence in the validity of rituals to some degree. While individual failures may be rationalized, they have some cognitive effect, and sometimes repeated or prominent failures can subtly erode both individual and collective belief in the efficacy of magical rituals. A Bayesian approach is particularly promising here, as it allows belief to be modeled as a matter of degree, illustrating how disconfirming evidence can be explained away by updating auxiliary beliefs while still producing a small but non‐zero erosion of confidence in the ritual itself. This, however, creates a deeper puzzle: If individual minds are updating in response to failure, why do these small doubts not aggregate over generations into collective skepticism and the eventual collapse of the ritual system? I suggest the answer lies not in the absence of belief revision, but in the cultural ecology in which those cognitive mechanisms are embedded. This ecology systematically inhibits the aggregation of doubt through several mutually reinforcing factors: the protective “design” of rituals, which often makes them inherently difficult to falsify; biased information transmission, such as the selective memory and underreporting of failures; and powerful social dynamics like pluralistic ignorance, where individuals mistakenly infer collective confidence from public conformity. In traditional societies, these effects are compounded by the lack of institutional structures for record‐keeping and critical discourse, which might otherwise allow disconfirming evidence to accumulate. Notably, this dynamic extends beyond classic anthropological notions of magic and is characteristic of instrumental reasoning in general [23].

Before proceeding, I shall briefly address the definitional issues surrounding “ritual,” a concept notoriously difficult to pin down in anthropology [24]. Eschewing protracted debates, I adopt a working definition that aligns with the intellectualist tradition: ritual as ineffective technology. By this, I refer to cultural practices in which participants intentionally attempt to achieve specific outcomes that are, from a modern scientific standpoint, objectively futile. This definition is justified not as a universal or emic account (indeed, “ritual” is sometimes defined as the opposite of instrumental action; see [25]), but as a heuristic device that foregrounds the instrumental dimension of ritual—specifically, the ways in which people use ritual action to try to influence concrete outcomes in the world [16]. It thus enables a focused analysis of what happens cognitively and socially when these attempts fail. Under this definition, “ritual” as a general category encompasses not only classic anthropological concepts such as magic and divination but also various forms of occultism and esoteric practices found in diverse human societies. This approach aligns with common contemporary understandings of ritual and deliberately excludes secular ceremonies such as weddings and sporting events [12]. Given this definition, “ritual failure” refers to instances where a ritual visibly fails to achieve its intended outcome rather than its social functions (cf. Hüsken's [26] distinction between “outcome oriented” and “procedure oriented” view of ritual failure). Examples include inaccurate divinatory verdicts, rainmaking rituals that do not bring precipitation, protective spells that do not prevent harm, and healing ceremonies that do not cure illness (Notice that this definition of ritual is significantly different from some recent psychological approaches to rituals that primarily emphasize their social and regulatory functions [27, 28]).

The rest of the paper is organized as follows. I start by surveying existing theories of rationalization of ritual failures in both anthropology and psychology (Section 2), and then examining the cognitive consequences of ritual failures (Section 3). Next, I introduce Bayesian belief updating and predictive processing as theoretical tools for modeling the maintenance of magical beliefs (Section 4). Finally, I analyze the barriers to cumulative skepticism arising from ritual failure and explore why rituals and the underlying beliefs in their efficacy persist in many traditional societies (Section 5).

## 2 Rationalization and Theories of Rationalization of Ritual Failure

Rationalization, in its broad sense, refers to the process of providing explanations or justifications for an outcome, particularly when the outcome is contrary to initial expectations. In this article, I use “rationalization” in a sense closely aligned with the concept of auxiliary hypotheses in the philosophy of science—namely, additional assumptions introduced to reconcile discrepancies between expected and observed outcomes [29]. Within this framework, rationalization serves to preserve—albeit not completely (see Sections 3 and 4)—belief in the efficacy of rituals despite observable failures. This usage differs from the narrower sense of rationalization commonly employed in psychology, which denotes the post‐hoc creation of beliefs or desires to retrospectively justify an action as rational [30]. While this psychological form of rationalization sheds important light on individual behaviors and motivations, my primary focus here is broader: I examine how cultural and epistemic systems rationalize ritual failures, the cognitive consequences of these rationalizations, and their implications for the persistence of beliefs and belief systems. In the sections that follow, I explore this phenomenon through two complementary lenses: anthropological theories, which document cultural strategies for rationalizing ritual failure, and psychological theories, which elucidate the cognitive mechanisms underpinning these practices.

### 2.1 Rationalization in Anthropology

Since the very beginning of modern academic anthropology, scholars have noticed that ritual failures tended to be explained (away) in many traditional societies. In his classic work *Primitive Culture*, Edward Tylor [10] offers one of the earliest systematic accounts of this phenomenon, framing it within his intellectualist perspective on magic and religion. Tylor writes:

By far the larger proportion [of the outcomes of magic], however, are what we should call failures; but it is a part of the magician's profession to keep these from counting, and this he does with extraordinary resource of rhetorical shift and brazen impudence…Has a daughter been born when he promised a son, then it is some hostile practitioner who has turned the boy into a girl; does a tempest come just when he is making fine weather, then he calmly demands a larger fee for stronger ceremonies, assuring his clients that they may thank him as it is, for how much worse it would have been had he not done what he did.

[10], p. 134

Tylor suggests that practitioners exhibit an “incapacity to appreciate negative evidence,” where a single success can outweigh multiple failures in their reasoning [10], Vol. 1, p. 136. For Tylor, this reflects a form of faulty causal attribution rooted in the human tendency to seek patterns and agency, a view that aligns with his broader evolutionary theory of cultural development from “savagery” to “civilization.” While Tylor's analysis has been critiqued for its ethnocentrism [8], his focus on rationalization as a cognitive strategy highlights an enduring feature of magical practices: the ability to deflect disconfirming evidence through ad hoc explanations.

This pattern of rationalization is vividly illustrated in later ethnographic work. Bronisław Malinowski [31], in his study of the Trobriand Islanders of Melanesia, describes how magic may persist despite observable failures:

First of all, magic is surrounded by strict conditions: exact remembrance of a spell, unimpeachable performance of the rite, unswerving adhesion to the taboos and observances which shackle the magician. If any one of these is neglected, failure of magic follows. And then, even if magic be done in the most perfect manner, its effects can be equally well undone: for against every magic there can be also counter‐magic…Thus the failures of magic can be always accounted for by the slip of memory, by slovenliness in performance or in observance of a taboo, and, last not least, by the fact that someone else has performed some counter‐magic.

[31], p. 66

To be sure, Malinowski's theory of magic is fundamentally functionalist (emphasizing the psychological comfort these rituals provide). However, his observations also reveal the cognitive strategy of preserving belief through auxiliary hypotheses—a point that resonates with Tylor's intellectualist insights.

Perhaps the most famous anthropological theory of rationalization comes from E.E. Evans‐Pritchard's [32] seminal work *Witchcraft, Oracles and Magic Among the Azande*. Evans‐Pritchard introduces the concept of “secondary elaboration,” whereby Zande practitioners and clients explain oracle failures through a range of ad hoc factors. He lists a number of examples:

The secondary elaborations of belief that explain the failure of the oracle attribute its failure to (1) the wrong variety of poison having been gathered, (2) breach of a taboo, (3) witchcraft, (4) anger of the owners of the forest where the creeper grows, (5) age of the poison, (6) anger of the ghosts, (7) sorcery, (8) use [of improper procedures].

[32],p. 155

For the Zande, the oracle's validity is never fundamentally questioned; instead, failures are absorbed into a web of auxiliary assumptions that shield the core belief from falsification. Evans‐Pritchard's list gives the impression that oracular success is almost miraculous: so many conditions must be met for divination to yield accurate information. The fragility of magical success has been noted by other ethnographers as well [33], for instance, documents various factors that could nullify a specialist's supernatural power, such as taboo violations or failure to respect one's master. The concept of “secondary elaboration” has been further articulated by Horton [5], who argues that it is a characteristic mode of reasoning in many traditional African religions.

More broadly, the tendency to invoke ad hoc explanations for ritual failures is so prevalent that most ethnographers and historians studying traditional religious practices have remarked on it to some extent. This is particularly evident when some divine agents are involved in the delivery of the desired ritual outcome. Commonly noted factors include the perceived insincerity of the participants [18], the capriciousness of the deities [34], the incompetence of ritual specialists [35], p. 80, and in the case of divination, the inherent limitations of human cognition in accurately interpreting divine signs [36]. However, these accounts typically address ritual failures in a piecemeal manner, lacking the systematic and coherent frameworks necessary for a comprehensive analysis of ritual failure rationalization. I now turn to major psychological theories that offer explicit cognitive perspectives on these pervasive patterns of belief maintenance.

### 2.2 Rationalization in Psychology

In psychology, though the term “rationalization” is often narrowly understood as a belief concocted after the action has been committed, it may be broadly understood as a cognitive process through which individuals provide justifications or explanations to reconcile discrepancies between expectations and outcomes, often to mitigate discomfort or preserve valued beliefs.

Early psychoanalytic theory describes rationalization as a subconscious defense mechanism that protects the ego from psychological threat. This process works by creating plausible, socially acceptable justifications for behaviors, beliefs, and attitudes that actually stem from unacceptable unconscious impulses [37]. Despite the decline of psychoanalysis in mainstream psychology, the “rationalization‐as‐defense” idea continues to inform therapeutic practices and psychological theories that address how individuals adapt to stress and maintain psychological equilibrium [38].

In contrast to psychoanalytic approaches that emphasized impulse and viewed rationalization as a primarily affective response to psychological tension, later psychological theories adopted a more explicitly cognitive perspective. Among these, Leon Festinger's [39] cognitive dissonance theory stands as one of the most influential explanations of rationalization in psychology. The core idea is that individuals experience a psychologically aversive state, or “dissonance,” when their beliefs, desires, and actions are inconsistent with one another. This discomfort creates a powerful motivation to restore consistency. Since an action already committed cannot be undone, this resolution is often achieved by adjusting one's beliefs or desires to retroactively justify the action—a process that is the essence of rationalization in this framework (Importantly, there have been recent replication efforts that cast doubt on the existence of cognitive dissonance [40]). In the context of ritual, because ritual performances often represent significant, public, and irreversible social and psychological commitments, participants who face a failed outcome cannot easily disavow their actions. Instead, to resolve the dissonance between their committed participation and the disconfirming evidence, they may be motivated to adjust their beliefs. For instance, they might rationalize the failure by concluding the ritual *did* work in an unseen way or that their faith was being tested, thereby preserving their core belief in the ritual system itself.

A related, yet distinct, psychological framework is motivated reasoning, which refers to the tendency for individuals to process information in ways that align with their pre‐existing beliefs, desires, or social identities [41]. Rather than being strictly a mechanism for reducing existing dissonance, motivated reasoning can also function proactively to prevent dissonance from arising in the first place. It achieves this by biasing how information is sought, interpreted, and evaluated to align with a desired conclusion. This process is not limited to protecting a positive self‐image but can be engaged to defend any cherished belief, often through powerful mechanisms like the confirmation bias, where individuals preferentially seek out and favor information that confirms their existing views while scrutinizing or dismissing contradictory evidence [42]. There has been much empirical research on motivated reasoning in domains such as political polarization [43], religious beliefs [44], vaccine hesitancy [45], and conspiracy thinking [46]. In all of these cases, individuals are more likely to scrutinize or reject evidence that contradicts their identity/desire‐relevant beliefs, while readily accepting evidence that confirms their worldview. In the context of ritual, motivated reasoning frames the rationalization of ritual failure as a psychological response to socially or existentially threatening outcomes. In this respect, it bears some resemblance to Freudian defense mechanisms in the psychoanalytic tradition, which similarly explain rationalization as a protective response to psychologically distressing contradictions. However, unlike the Freudian account, motivated reasoning is grounded in contemporary cognitive psychology and emphasizes goal‐directed cognition rather than unconscious repression.

Before introducing an alternative computational framework to explain these phenomena, the next section (Section 3) will first offer a deeper exploration of the cognitive consequences of ritual failures. Following this more detailed analysis, Section 4 will then introduce an alternative grounded in Bayesian models of cognition. This approach, which reframes rationalization as a core feature of predictive processing, provides a more fundamental explanation for the patterns of belief maintenance discussed here.

## 3 The Cognitive Consequence of Ritual Failures

Having examined both anthropological and psychological approaches to rationalization, we are now in a good position to consider their implications for understanding how ritual failures shape belief systems. Given the instrumental nature of rituals as defined earlier, individual ritual instances must have failed with substantial frequency. The exact failure rate likely depends on the nature of the desired outcome: traditional fetal sex prognostication, for instance, presumably fails 50% of the time [19], whereas rainmaking rituals, which depend on complex meteorological variables beyond human control, likely failed at a much higher rate [18]. In cases where the expected outcome is ambiguous or difficult to verify, failures may go unnoticed or be open to reinterpretation. However, in cases where failure is clearly observable such as an unfulfilled prophecy or a failed healing ritual, the cognitive salience tends to be more pronounced.

Here, it is worth emphasizing that the outcomes of rituals do matter—sometimes tremendously. Granted, ritual failures in many traditional societies differ from the failed prophecy as described in Festinger et al. (1956) in that many participants in ritual practices are well aware that specific ritual instances may fail based on past experiences (surely, many instrumental rituals must have failed at considerable frequencies), but this does not mean that ritual outcomes are taken lightly. Rituals can be very costly [47], and in high‐stakes cases such as rainmaking, ritual specialists may face severe consequences for failure. Ethnographic accounts describe instances where failed rainmakers were beaten [48] or even executed [49, 50]. Even in more routine ritual contexts, failures do not simply go unnoticed; instead, they often trigger efforts to distinguish between competent practitioners and frauds, as numerous ethnographic records have demonstrated. Thus, rather than being dismissed as inconsequential, ritual failures frequently serve as salient cognitive events, prompting individuals to search for underlying explanations [51].

As discussed in Section 2, the prevailing impression from the psychological literature is that individuals typically rationalize such failures in ways that preserve their core belief in ritual efficacy. A more subtle and often overlooked point, however, is that many scholars either explicitly or implicitly assume that people's confidence in the validity of rituals remains *wholly* unaffected by failure. Indeed, ethnographic and historical records are replete with descriptions suggesting the perceived invulnerability of ritual efficacy [5], for instance, describes traditional African religious systems by stating, “…the client never takes his repeated failures as evidence against the existence of the various spiritual beings named as responsible for his plight, or as evidence against the possibility of making contact with such beings as diviners claim to do.” Similarly, Hsu [52] notes that the authority of the traditional Chinese healer remains “secured from any doubt” even in cases of failed healing. Evans‐Pritchard [32] observed a similar phenomenon among the Azande, commenting that “the fact that the oracle is wrong when it is interfered with by some mystical power shows how accurate are its judgments when these powers are excluded.”

The historian Keith [53] likewise highlights the resilience of magical beliefs in medieval Europe, specifically stating that failures do not matter because they can be explained away:

…once their initial premises are accepted, no subsequent discovery will shake the believer's faith, for he can explain it away in terms of the existing system. Neither will his convictions be weakened by the failure of some accepted ritual to accomplish its desired end, for this too can be accounted for…

Thomas concludes that the reaction against magic could never come from the “cumulative resentment of disappointed clients” [53], p. 767, but had to arise from outside the system altogether—a point echoed in explanations for the decline of astrology [54] and alchemy [55].

We get the same impression from the psychological theories of rationalization when applied to ritual failures. This is most apparent in Festinger's theory of cognitive dissonance which he uses to account for the maintenance of religious cults, and both Freudian psychoanalytic defense mechanisms and various motivational theories suggest that individuals are capable of resisting belief modification to such an extent that disconfirming evidence not only fails to weaken their convictions but, in some cases, even strengthens them [56]. This line of research often treats rationalization dismissively as mere “post hoc” reasoning [57, 58], implying that the belief change is a consequence of an action, rather than its cause.

However, such conclusions may overstate the resilience of belief systems, particularly at the individual level. Since rituals are instrumental activities, the way people mentally process their outcomes should, at least in part, be subject to the same general psychological principles (such as operant conditioning) that govern other goal‐directed behaviors [16]. When rituals produce the expected outcome, belief in their efficacy is reinforced; conversely, ritual failure constitutes negative feedback, which under normal cognitive conditions should lead to some adjustment in expectations or behavior. The key question, then, is under what conditions this feedback meaningfully affects belief in ritual efficacy, and to what extent.

There is some evidence showing that rituals and the belief systems that sustain them are not entirely immune to skepticism, especially when failures are particularly dramatic [59]. For example, scholars have attributed the decline of the Ghost Dance movement among the Cherokee in the 19th century to unfulfilled predictions. Prophecies associated with the movement, such as a hailstorm that was supposed to destroy the earth and threats of death to nonbelievers never materialized, leading to disillusionment and eventual abandonment of the practice [60], p. 113. Similarly, in the Millerite movement in 19th‐century America, followers believed that the world would end on a specific date based on prophetic calculations. When the prophecy failed—a day known as the “Great Disappointment“—the movement experienced profound turmoil, with many members leaving in disillusionment [61]. Even in Festinger et al.'s (1956) classic case study, some followers ultimately lost faith and left the cult after experiencing repeated disconfirmation.

In more mundane contexts where rituals are performed to address everyday concerns, both practitioners and participants are often aware that specific ritual instances may fail. In such cases, Boyer [22] makes an important distinction between “public certainty” and “private doubt.” He argues that while people rarely openly challenge the validity of divinatory techniques (though such dissent does occasionally occur; see [62], p. 70), private reflections on ritual failures can and do weaken confidence. Individuals may not explicitly renounce their beliefs, but each instance of failure accumulates in their cognitive assessments. Lewis [63] makes a similar point in his study of healing rituals among the Gnau of Papua New Guinea: despite the public performance of confidence and communal unity, ritual participants privately reflected on the efficacy of the ritual and questioned whether it was truly working, revealing a likely gap between collective expression and individual cognition. Moreover, it is important to recognize the heterogeneity within belief systems [64]. Not all individuals hold their convictions with equal strength: some are more tentative or half‐hearted in their adherence. For these individuals, ritual failures are more likely to exert significant cognitive effects, gradually diminishing their belief.

Similar patterns can be observed in contemporary religious contexts, particularly in the domain of prayer. Luhrmann [65] shows that doubt is a pervasive undercurrent in evangelical Christianity. Failed prayers often generate emotional distress and, in some cases, lead to private disillusionment that results in withdrawal from religious organizations (p. 289). Through in‐depth interviews, LeCount [59] similarly shows that unanswered prayers can lead some Christians to question their relationship with God, or even doubt His existence. Zuckerman [66] also observes that while many Christian believers are able to rationalize failed prayers, for a minority, these failures serve as catalysts for apostasy.

Why, then, do most ethnographic and historical accounts emphasize the resilience, if not the invulnerability, of belief in the efficacy of rituals (and prayers, insofar as they can be considered rituals), rather than their erosion? One explanation may be that scholars have historically favored documenting the exotic, the striking, and the seemingly irrational [67], p. 8. But perhaps more importantly, changes in belief following ritual failures are often imperceptible in the short term. These changes may be subtle, gradual, and relatively small in magnitude, especially when measured against the strong prior commitment to the belief system that sustains ritual efficacy. In other words, it is not that ritual failures have no cognitive effect; rather, their impact is often too weak to produce immediate, observable changes in behavior or discourse. This does not mean the effect is negligible; only that it may be difficult to detect ethnographically. In what follows, I examine how the Bayesian framework and the theory of predictive processing can help us better understand the cognitive consequences of ritual failure.

## 4 Rationalization of Ritual Failure From the Perspective of Cognitive Science: Bayesianism and Predictive Processing

### 4.1 Bayesian Belief Updating and Rationalization

In the broadest sense, Bayesian belief updating refers to the process by which an agent revises their beliefs in light of new evidence, proportionally adjusting their confidence based on how likely the evidence is under competing hypotheses. As such, Bayesian inference provides a particularly suitable framework for understanding both the direction and the magnitude of belief change in response to new information.

In a recent proposal [68], Gershman suggests that the apparent resilience of some beliefs or belief systems in the face of disconfirming evidence may be understood as *rational* after all. Specifically, in cases where prior beliefs are especially strong—such as those underpinning conspiracy theories, political ideologies, or religious worldviews—it is Bayesian‐rational to invoke additional explanatory factors, known as *auxiliary hypotheses* in the philosophy of science [69] to account for anomalous or unexpected outcomes. For example, when a rainmaking ritual fails to produce rain, adherents may explain the failure not by questioning the ritual's validity but by invoking external interference, such as the presence of a taboo‐breaker in the community or insufficient ritual purity. Key to this analysis is the relative strength of the prior probability assigned to the central hypothesis versus that of the auxiliary hypotheses. If the prior for the central hypothesis is particularly strong, then it will be robust to disconfirmation by effectively shifting explanatory responsibility to the auxiliary hypothesis—a process Gershman [68] refers to as “pushing blame onto the auxiliary.” This idea builds on earlier efforts to incorporate auxiliary hypotheses into Bayesian models of reasoning [70, 71].

Gershman's proposal is part of a broader scholarly movement that re‐examines phenomena often attributed to motivated reasoning or confirmation bias through the lens of Bayesian inference. This body of work argues that patterns of belief updating that appear “biased“ may in fact be consistent with a normatively rational process. As Tappin and Gadsby [72] argue in their review, much of the empirical evidence purporting to show violations of Bayesian principles is often unconvincing or can be explained by more sophisticated Bayesian models. For instance, Cook and Lewandowsky [73] use Bayesian networks to demonstrate that belief polarization regarding climate change, a classic case of “irrational“ updating, can be simulated within a fully rational framework by accounting for individuals’ prior beliefs about the trustworthiness of scientists. Similarly, Tappin, Pennycook, and Rand [74] show that belief in political information can be better explained by a Bayesian model than by politically biased processing. This perspective suggests that what is often labeled as motivated reasoning may be the result of rational agents applying different, but internally coherent, likelihoods to new information. Crucially, this Bayesian perspective aligns with a key empirical finding about persuasion: when individuals encounter challenging information, they often update their beliefs by a very small, incremental amount rather than refusing to change their minds entirely. In his extensive analysis of persuasion experiments, Coppock [75] finds this pattern of small, parallel belief change to be the norm. This aligns perfectly with a key prediction of the present model: that each failure produces a small but non‐zero erosion of confidence. Coppock [75] explicitly notes that this pattern is consistent with Bayesianism, even if it can be empirically difficult to distinguish from some forms of motivated reasoning.

In the case of ritual failure, the *central hypothesis* is that rituals are effective instrumental actions. The *auxiliary hypotheses* include various potential sources of interference, such as the incompetence of the ritual specialist, moral impurity among participants, or unfavorable cosmological conditions. There is considerable evidence suggesting that the prior probability of the central hypothesis may be especially strong in many cultural contexts, in part because the validity of rituals is embedded in a broader web of interlocking, shared cultural assumptions. Denying the efficacy of ritual, therefore, risks threatening not just a single belief but the coherence of an entire worldview [5, 32]. As Rappaport [76] notes, rituals are often treated as unfalsifiable in principle because they are believed to be sanctioned by deities who, by definition, do not err. In such contexts, questioning the validity of ritual entails questioning foundational beliefs about the ontological and causal structure of the world. Consequently, the strength of the central belief compels a Bayesian reasoner to attribute failure to more peripheral, less‐entrenched factors like human error or situational irregularities. As such, the rationalization of ritual failure from a Bayesian perspective may be viewed as the epistemic move of increasing the posterior probability of one or more auxiliary hypotheses that account for the failure, which enables the preservation of the core belief while still incorporating new evidence.

I hasten to add, however, that this is not to say the belief in ritual efficacy is invulnerable. A key insight from Gershman [68]—consistent with earlier Bayesian analyses of hypothesis testing [70, 77]—is that while auxiliary hypotheses can absorb much of the blame, the posterior probability of the central hypothesis still decreases in the face of disconfirmatory evidence. Let us denote the central hypothesis as *h*, the auxiliary hypothesis as *a* (for simplicity, assuming only one auxiliary hypothesis), and the observed disconfirmatory data as *d*. Strevens [71] derives the following expression for the posterior probability of *h* given *d*:

$$P\left(h|d\right)=\frac{1-P\left(a|h\right)}{1-P\left(a|h\right)P\left(h\right)}P\left(h\right)$$

This equation has several intuitive properties, but one is particularly important: the posterior probability of the central hypothesis *always* decreases in response to disconfirming data. This is because the multiplier $\frac{1-P\left(a|h\right)}{1-P\left(a|h\right)P\left(h\right)}$ is always less than one, which guarantees that P(h|d)<P(h), provided that P(h|d) and P(h) are between 0 and 1. Notice that such decrease may be very small when the prior belief of the central hypothesis P(h) is large, implying that the central belief (e.g., that ritual actions are effective) can appear rather resistant to counter‐evidence.

We thus arrive at a satisfying theory‐observation match: Bayesian theory predicts that it is entirely rational for individuals to update their belief in ritual efficacy only minimally in the face of ritual failures, when the prior belief is strong. This prediction accords with ethnographic and historical observations that beliefs in ritual efficacy tend to be resistant to disconfirmation, though small shifts in belief do occasionally occur. But does Bayesianism accurately reflect our psychological reality? While this question has sparked considerable debate [78, 79, 80], there are reasons to believe that human cognition is at least approximately Bayesian. From an evolutionary perspective, natural selection should select for cognitive mechanisms that deal with uncertainty adaptively, and in many analytic frameworks, systems that implement Bayesian algorithms are among the most rational in managing uncertainty [81]. Computationally speaking, even if the brain is not explicitly representing probability values or densities, it is still combining prior knowledge with new evidence in ways that respect (by approximating) probabilistic Bayesian inference [82]. Bayesian models of cognition have been extensively applied in numerous cognitive domains, including attention [83], categorization [84], language processing [85], concept learning [86], inductive/deductive reasoning [87], and theory of mind [88]. Crucially, this approximation may be achieved by the brain operating as a computationally efficient but error‐prone sampler [89].

### 4.2 Predictive Processing and Rationalization

More recently, the “predictive processing” framework has gained significant traction in cognitive science [90, 91] and has been proposed as a neurocognitive mechanism for implementing approximately rational Bayesian belief updating [92]. Pioneered by Friston [93] and his colleagues, predictive processing views the brain as a hierarchical generative model that continuously produces predictions about sensory input. By comparing incoming sensory data with these internally generated predictions, the brain attempts to minimize the difference known as “prediction error” either by updating its internal model or by acting to change the external world. As Hohwy [91] points out, if a system persistently minimizes prediction error, then it will, in effect, approximate Bayesian inference. This is because minimizing prediction error entails continuously weighting prior expectations against new sensory input according to their respective precision, a measure roughly corresponding to their estimated reliability, much like Bayesian updating weighs priors and likelihoods.

In some sense, predictive processing is a more ambitious theory in that it aspires to provide a unified account of cognition and action. It is firmly grounded in physiology and neuroscience [94, 95] but has also been extended to macro‐level phenomena such as human culture and social cognition [96]. Predictive processing also provides the theoretical language to bridge the gap between the abstract mathematics of Bayesianism and the reality of subjective human experience. In this framework, people's understanding of instrumental rituals may be conceptualized as a generative model, a mental model that specifies expectations about the likelihood of different outcomes (e.g., success vs. failure). This model is continuously updated as new information about ritual efficacy is processed by the brain. If rituals produce strong top‐down predictions about how the world should respond (i.e., rain should follow a rainmaking ritual), then failed outcomes create significant prediction error, forcing the cognitive system to reduce this error by updating the generative model.

In the context of ritual failure, belief updating via predictive processing resembles cognitive dissonance in that both involve a mismatch between expectation and observed outcome. Indeed, there have been efforts to integrate cognitive dissonance theory within the broader framework of predictive processing [97]. The two frameworks, however, differ in important ways. Cognitive dissonance theory posits that inconsistencies between beliefs and outcomes generate psychological discomfort (dissonance), which in turn motivates individuals to reduce the inconsistency, often by rationalizing the failure or reaffirming the original belief. By contrast, predictive processing approaches this mismatch computationally: the brain attempts to resolve prediction error regardless of whether the inconsistency is consciously experienced as discomfort. For example, when a healing ritual fails to cure an illness, the brain does not need to feel “dissonance“ per se; it may automatically adjust its generative model by downgrading the reliability of the ritual specialist or considering a secondary factor (e.g., insufficient ritual purity), all in service of minimizing error in its internal model.

From a predictive processing perspective, rationalization via the invocation of auxiliary hypotheses can be understood as a byproduct of the system's effort to minimize prediction error while maintaining internal coherence. One important consequence of invoking auxiliary hypotheses to explain away failed outcomes is that the generative model becomes increasingly complex. That is, as individuals retrospectively search for causes to account for ritual failures, they tend to introduce new conditions or caveats under which the ritual is expected to work. Over time, this results in a proliferation of auxiliary assumptions—such as specific timing, environmental purity, or ritual specialist competence—which collectively inflate the models complexity. In this sense, rationalization contributes to an ever‐growing explanatory framework [51], helping to account for the often elaborate and excessive cultural beliefs regarding the requirements and procedures for the proper functioning of rituals in traditional societies [98].

An important implication of the updating of the generative model in predictive processing is that, because such updating occurs at a fundamental cognitive level that automatically performs epistemic computations, cognitively normal human individuals will inevitably modify their internal representations of the external world to some extent, including their beliefs about instrumental rituals. In Bayesian terms, even if the mind functions merely as a sampler which does not represent all hypotheses simultaneously [92], it is highly unlikely that the hypothesis “*ritual does not work*,” or more provocatively, “*the deities we attempt to communicate with do not exist*” is entirely absent from the mind (Though there have been anthropological arguments that individuals in traditional societies typically express little theoretical interest in general principles or the elements of their world [99]). Rather, such hypotheses (logical opposites of the central hypotheses asserting ritual efficacy) likely exist as low‐probability priors in our generative model of the world.

Disillusionment with ritual efficacy can, in some cases, be abrupt and dramatic, particularly when the theoretical foundations that support ritual belief are disrupted by strong external forces (e.g., when a supernatural, animistic worldview is suddenly replaced by a materialist one [18]). More commonly, however, growing skepticism toward rituals—or, equivalently, a weakening of confidence in their efficacy—unfolds gradually. This process typically involves a continuous reweighting of competing hypotheses in response to prediction errors, such as failed ritual outcomes, accumulated over the course of an individual's life. In most cases, prediction errors are absorbed by peripheral auxiliary beliefs, which are themselves part of the generative model. These include explanations that attribute failure to human error, improper timing, or ritual impurity. As a result, the core belief in the efficacy of ritual tends to remain intact, or only slightly weakened, even as confidence varies across individuals. In a Bayesian framework, this variation can be represented by assigning different degrees of belief (modeled as real numbers between 0 and 1) to the central hypothesis. Indeed, there is likely substantial heterogeneity in individuals’ confidence regarding the efficacy of specific ritual practices [17].

Importantly, as suggested by the drift‐diffusion model of decision‐making in perception research [100], there may be cognitive thresholds that the cumulative effects of disconfirmation (though often constrained; see Section 5) must exceed before individuals experience overt, conscious doubt. Only after such thresholds are crossed might belief revision manifest behaviorally, for instance in the refusal to rely on rituals for particular purposes. Thus, even in tightly knit cultural systems with strong ritual commitments, belief can shift subtly and incrementally at the cognitive level, even in the absence of explicit skepticism or deliberate reflection.

## 5 The Persistence of Rituals in Light of Cognitive Science: (Lack of) Cumulative Skepticism, Information Loss, and the Protective Design of Rituals

Despite their objective ineffectiveness in producing causal outcomes, instrumental rituals remain widespread and enduring across human societies. This section develops the argument that cumulative skepticism—the slow accumulation of doubt across individuals and (more importantly) generations that might ultimately lead to large‐scale belief revision or the collapse of entire explanatory systems—is typically absent in traditional societies. While individuals do update their beliefs during their lifetimes, the conditions required for these updates to aggregate into population‐level change are rarely met. This failure stems from multiple sources: the nature of predictive cognition, biased information transmission, the “design” of rituals that pre‐emptively shield them from empirical disconfirmation and the lack of institutional structures that support epistemic consolidation and collective doubt (This broader pattern connects to what Sterelny [101] has described as a paradox of instrumental action. On the one hand, traditional societies have demonstrably solved complex instrumental problems such as detoxifying poisonous plants [102]—cases that require the gradual accumulation of evidence across generations under highly noisy conditions. On the other hand, medical practices that are ineffective, and in some cases actively harmful, have often persisted for centuries [103]. Why cumulative progress succeeds in some instrumental domains but not in others remains an open question for future theoretical and empirical study. One possible factor is intuitive plausibility: practices that “feel” effective may be more likely to spread and persist regardless of their objective efficacy. Singh [104] has proposed that certain practices function as cultural “super‐attractors,” whose intuitive appeal stabilizes them even in the absence of real efficacy. More generally, Hong and Henrich [17] argue that the persistence or abandonment of technological practices reflects the interplay of intuitive plausibility, objective effectiveness, and transmission dynamics. From this perspective, the contrast between detoxification and medicine may be partly explained by the alignment [or misalignment] between what seems compelling to human intuition and what actually works).

### 5.1 Individual Belief Updating Without Cumulative Skepticism

Both the Bayesian and the predictive processing framework suggest that human belief systems are constantly updated in response to discrepancies between expectations and observed outcomes. However, in practice, the cognitive effect of ritual failures is often limited. Strong prior beliefs, often deeply entrenched and socially reinforced, constrain how much weight is given to new evidence. As discussed earlier, prediction errors are typically not attributed to flaws in the ritual itself, but are instead explained away via auxiliary beliefs such as blaming the ritual specialist's incompetence, the impurity of the participants, or the inauspiciousness of the timing. These adjustments allow the core belief in the ritual's efficacy to remain largely (though not entirely) intact.

Over time, repeated exposure to such errors may lead to a gradual weakening of private confidence. However, this erosion of belief often remains below the threshold of conscious awareness or social articulation. As Clark [82] suggests, even though the brain operates on probabilistic principles, it typically produces unitary and coherent perceptual experiences. That is, while multiple hypotheses with varying degrees of confidence may be represented at the sub‐personal level, only one interpretation is selected for conscious experience and action. This helps explain why ritual failures do not typically result in overt disbelief or deliberate questioning of the ritual system. At the subjective level, the brain favors stable and actionable interpretations such as attributing failure to ritual impurity or poor timing over probabilistic reflections like “there is a 5% chance that the ritual does not work.”

Even in cases where individuals are fully aware of the uncertainties involved, they may continue to behave as if they believe in ritual efficacy, not necessarily because their confidence is high, but because the cost–benefit calculus still favors participation. Rituals are often low‐cost, socially normative, and potentially beneficial if effective, whereas the perceived risks of nonperformance such as social disapproval or supernatural punishment are significant. Additionally, communal pressures, including coercive mechanisms to suppress dissent [62], p. 70, further discourage open expressions of doubt. As a result, public behavior does not necessarily reflect private skepticism.

In short, while belief updating at the individual level is a universal cognitive process, it does not automatically aggregate into cumulative skepticism at the population level. The failure to achieve cumulative skepticism is not due to the absence of cognitive mechanisms for belief revision, but to the cultural ecology in which those mechanisms are embedded. In modern societies, the existence of systems for record‐keeping, critical discourse, and empirical evaluation allows disconfirming evidence to accumulate and circulate, facilitating widespread belief revision. Traditional societies, by contrast, often lack such epistemic infrastructure. As a result, individual doubts remain isolated, rarely converging into collective disillusionment or paradigm‐level change.

### 5.2 Information Loss and the Underreporting of Failure

From the perspective of cultural transmission, one of the primary barriers to cumulative skepticism is the systematic loss or distortion of information about ritual outcomes. In most traditional societies, there are no formal mechanisms such as written records, systematic observations, or independent verification for documenting ritual success or failure. Instead, ritual efficacy is remembered and transmitted through oral traditions and personal anecdotes, both of which are subject to selective recall and social filtering.

Failed rituals are often forgotten, downplayed, or reinterpreted, while successful or ambiguous cases are more likely to be remembered, retold, and socially endorsed. This creates a transmission bias that disproportionately favors narratives of ritual efficacy [14, 15, 18, 19]. As a result, each generation receives a skewed sample of information, in which failures are underrepresented and successes overrepresented.

Moreover, individuals draw not only from testimonial accounts of ritual outcomes, but also from the observed behavior of others when forming or updating beliefs [17, 105]. When people see others continuing to perform rituals, they infer that those individuals still believe in their efficacy. This inference is often mistaken: others may be acting out of conformity, habit, or precaution, not conviction. But because private beliefs are invisible, and behavior is publicly observable, this creates a powerful illusion of consensus. This phenomenon is a Bayesian variant of pluralistic ignorance [106]: everyone sees ritual participation and assumes that belief remains strong, while in reality many may privately harbor doubts. Because of this misperception, individuals have little reason to challenge the system, and their own doubts are further muted by the apparent confidence of others. These intertwined biases in personal memory and social observation effectively shield the ritual belief system from accumulating disconfirmation.

### 5.3 The Design of Rituals, Cultural Evolutionary Implications, and Contrasts With Modern Science

Another important factor that contributes to rituals’ resilience against apparent empirical disconfirmation is the design of rituals themselves. Often, rituals and ritual systems are not structured for falsifiability in the sense that they are not designed to be tested, replicated, or subjected to institutionalized skepticism, echoing comments from classic anthropology and sociological work that traditional thought systems are “circular” [107] or “closed” [5]. Instead, they are protected by sacred narratives or theological doctrines that prohibit questioning core tenets: for example, the moral condemnation of disbelief in many monotheistic traditions, or posited intelligent agents (e.g., deities or spirits) who are said to conceal themselves deliberately from scrutiny, making empirical disconfirmation impossible [108]. As a result, belief in ritual efficacy persists not only because it is constantly reaffirmed by successful outcomes, but also because the conditions required for its collapse—sustained, visible, and socially transmitted doubt—rarely materialize.

From a cultural evolutionary perspective, these design features do more than simply shield ritual systems from empirical scrutiny—they may also confer selective advantages in the competitive landscape of cultural transmission [104]. Ritual systems that incorporate flexible, combinable auxiliary explanations are likely to be more robust under conditions of uncertainty, more adaptive to environmental and social variability, and more resistant to abandonment in the face of occasional failure. In effect, cognitive mechanisms of rationalization operate not only at the level of individual belief maintenance but also as stabilizers of cultural lineages, reducing the likelihood that disconfirming evidence cascades into population‐level collapse. For example, major Abrahamic religions have historically demonstrated remarkable resilience across centuries, in part due to rich theological resources that allow believers to reinterpret disappointment or failure within an encompassing explanatory framework [109]. Over time, such systems may outcompete less adaptable alternatives, contributing to their broad distribution and persistence across human societies.

This interplay between cognitive design and cultural transmission raises important contrasts with modern epistemic systems, particularly science. While ritual belief systems tend to persist through small, local parameter updates that preserve the core generative model, one of the major achievements of modern science has been its institutional capacity to discard entire generative models when faced with sufficient disconfirming evidence [17]. In the language of predictive processing, such worldview shifts involve radical updates to the underlying generative models that structure perception, action, and inference. These changes are not merely adjustments to specific beliefs, but constitute reorganizations of how reality is cognitively represented.

Another key mechanism by which scientific institutions attempt to resist the spread of ineffective or pseudoscientific practices is the establishment of explicit standards for empirical testing. Among the most powerful tools in this regard is the randomized controlled trial (RCT), a methodological design that isolates causal effects by randomly assigning participants to treatment and control groups. By controlling for confounding variables and accounting for phenomena such as the placebo effect where improvements result from expectations rather than the treatment itself [110, 111], RCTs offer a rigorous framework for determining whether a ritual, intervention, or treatment genuinely produces its intended outcomes. In doing so, they impose a level of epistemic discipline that makes it significantly harder for ineffective practices to persist on the basis of anecdote, tradition, or social reinforcement alone. While not immune to misuse or misinterpretation, the RCT remains a cornerstone of modern science's capacity to filter out spurious causal claims and to distinguish between action and efficacy.

### 5.4 Predictions, Empirical Tests, and Future Directions

The theoretical framework advanced in this paper contains multiple components ranging from cognitive mechanisms of belief updating to sociocultural dynamics of information transmission, which jointly contribute to the persistence of ritual systems in the face of repeated failure. As a result, the account generates a wide array of empirically testable predictions. Some of these predictions are relatively broad, offering general expectations about the direction of belief change under certain conditions; others are more precise, specifying how particular cognitive or cultural variables should modulate the effects of ritual failure. Below, I outline several predictions and offer concrete suggestions for future research to assess them.

Prediction 1: Partial belief updating following ritual failure. Consistent with Bayesian models and the predictive processing framework, exposure to failed ritual outcomes should lead to partial, but not complete reductions in confidence regarding ritual efficacy. The magnitude of belief updating is expected to depend on the strength of the prior belief and the availability of auxiliary explanations. This prediction can be tested through experimental paradigms that simulate ritual failures and measure changes in participants’ reported or inferred confidence levels over time.

Prediction 2: Belief erosion often occurs below the level of conscious awareness. Rather than generating overt dissonance, repeated ritual failures lead to subthreshold adjustments in internal generative models. This erosion may not be articulated verbally or even consciously registered, but could be detectable via implicit measures such as hesitation, reduced investment, or physiological responses. This prediction stems from the predictive processing account, which emphasizes subconscious model updates rather than explicit reflection.

Prediction 3: Ritual systems with more generative auxiliary space exhibit greater cultural stability. Belief systems that permit a wide, flexible, and combinable set of auxiliary explanations (e.g., moral pollution, improper timing, divine will) are predicted to better absorb disconfirming evidence and thus exhibit greater cultural stability. The more expansive and adaptable a system's auxiliary hypotheses, the more resistant it will be to collective skepticism and abandonment. In this way, cognitive flexibility directly contributes to the long‐term persistence of ritual systems.

These predictions offer a roadmap for future empirical work at the intersection of cognitive science and anthropology. Experimental studies using ritual simulations or narrative stimuli could test how people respond to ritual failures under different belief priors and explanatory conditions. Implicit measures and neurophysiological tools could help identify subthreshold belief updating. Cross‐cultural comparisons and historical analyses can assess whether auxiliary‐rich systems do in fact exhibit greater stability or slower belief attrition. Finally, computational modeling, particularly hierarchical Bayesian or drift‐diffusion models, could simulate the gradual weakening of belief over repeated prediction errors. Systematic empirical investigations along these lines would not only test the plausibility of the proposed mechanisms, but also contribute to broader theories of belief change, cultural evolution, and epistemic resilience.

## 6 Conclusion

I have argued that the persistence of belief in ritual efficacy, even in the face of failure, is best understood not as a failure of reasoning but as a consequence of how human cognition—especially under Bayesian and predictive processing frameworks—manages error and uncertainty. Ritual failures are not ignored, rather, they are rationalized through auxiliary explanations that maintain the coherence of core beliefs. Over time, this generates a gradual erosion of confidence, often subtle and uneven, but rarely sufficient to trigger public skepticism or systemic change. What makes ritual belief particularly resilient is not the strength of conviction alone, but the structure of the cultural and epistemic environments in which these beliefs are embedded. In the absence of mechanisms for aggregating disconfirmation such as formal record‐keeping, institutional critique, or controlled testing, individual doubts remain fragmented and socially inert. At the societal level, ritual systems thus persist not because they are shielded from failure, but because they are designed in ways that make failure difficult to accumulate, share, or act upon collectively.

## Data Availability

The author has nothing to report.
